# Exploring anterograde memory: a volumetric MRI study in patients with mild cognitive impairment

**DOI:** 10.1186/s13195-016-0190-1

**Published:** 2016-07-30

**Authors:** N. Philippi, V. Noblet, E. Duron, B. Cretin, C. Boully, I. Wisniewski, M. L. Seux, C. Martin-Hunyadi, E. Chaussade, C. Demuynck, S. Kremer, S. Lehéricy, D. Gounot, J. P. Armspach, O. Hanon, F. Blanc

**Affiliations:** 1 Department of Neurology, University Hospital of Strasbourg, Neuropsychology Unit, Strasbourg, France; 2 University of Strasbourg, CNRS, ICube laboratory, FMTS, Strasbourg, France; 3 University Hospital of Strasbourg, Centre Mémoire Ressources et Recherche, Strasbourg, France; 4 Department of Geriatrics, University Hospital of Strasbourg, Strasbourg, France; 5 Department of Geriatrics, Broca Hospital, Assistance Publique-Hôpitaux de Paris, Paris, France; 6 Department of Radiology, University Hospital of Strasbourg, Strasbourg, France; 7 Department of Neuroradiology, Groupe Hospitalier Pitié-Salpêtrière, Assistance Publique-Hôpitaux de Paris, Paris, France; 8 UPMC Paris 6—Inserm U1127, CNRS 7225, Institut du Cerveau et de la Moelle (ICM), Centre de NeuroImagerie de Recherche (CENIR), Paris, France; 9 Paris Descartes University, Sorbonne Paris Cité, EA4468 Paris, France

**Keywords:** Memory test FCSRT, DMS-48, Voxel-based morphometry, Mild cognitive impairment, Alzheimer’s disease, Medial temporal lobe, Hippocampus

## Abstract

**Background:**

The aim of this volumetric study was to explore the neuroanatomical correlates of the Free and Cued Selective Reminding Test (FCSRT) and the Delayed Matching-to-Sample—48 items (DMS-48), two tests widely used in France to assess verbal and visual anterograde memory. We wanted to determine to what extent the two tests rely on the medial temporal lobe, and could therefore be predictive of Alzheimer’s disease, in which pathological changes typically start in this region.

**Methods:**

We analysed data from a cohort of 138 patients with mild cognitive impairment participating in a longitudinal multicentre clinical research study. Verbal memory was assessed using the FCSRT and visual recognition memory was evaluated using the DMS-48. Performances on these two tests were correlated to local grey matter atrophy via structural MRI using voxel-based morphometry.

**Results:**

Our results confirm the existence of a positive correlation between the volume of the medial temporal lobe and the performance on the FCSRT, prominently on the left, and the performance on the DMS-48, on the right, for the whole group of patients (family-wise error, *P* < 0.05). Interestingly, this region remained implicated only in the subgroup of patients who had deficient scores on the cued recall of the FCSRT, whereas the free recall was associated with prefrontal aspects. For the DMS-48, it was only implicated for the group of patients whose performances declined between the immediate and delayed trial. Conversely, temporo-parietal cortices were implicated when no decline was observed. Within the medial temporal lobe, the parahippocampal gyrus was prominently involved for the FCSRT and the immediate trial of the DMS-48, whereas the hippocampus was solely involved for the delayed trial of the DMS-48.

**Conclusions:**

The two tests are able to detect an amnestic profile of the medial temporal type, under the condition that the scores remain deficient after the cued recall of the FCSRT or decline on the delayed recognition trial of the DMS-48. Strategic retrieval as well as perceptual/attentional processes, supported by prefrontal and temporo-parietal cortices, were also found to have an impact on the performances. Finally, the implication of the hippocampus appears time dependent, triggered by a longer delay than the parahippocampus, rather than determined by the sense of recollection or the encoding strength associated with the memory trace.

**Electronic supplementary material:**

The online version of this article (doi:10.1186/s13195-016-0190-1) contains supplementary material, which is available to authorized users.

## Background

Reliable markers are needed to detect early stages of Alzheimer’s disease, which is the most common cause of dementia and represents a challenge for both diagnosis and drug development. Even though new criteria for Alzheimer’s disease include the use of structural and molecular biomarkers [1, 2], cognitive symptoms remain the core feature of disease onset and a clinical examination including psychometric assessment still constitutes the first step of the diagnosis. More particularly, before performing high-cost and/or invasive examinations, the diagnosis requires clinical markers able to screen patients at risk of Alzheimer’s disease in a large population of patients with cognitive complaints. Mild cognitive impairment (MCI) [3] constitutes an appropriate condition for early detection of Alzheimer’s disease because it is a transitional stage between normal cognition and dementia, during which activities of daily living are still preserved. This condition, and more particularly the amnestic subtype (aMCI) [4], includes prodromal stages of Alzheimer’s disease [5]. Indeed, impaired memory is one of the earliest manifestations of typical Alzheimer’s disease, associated with the presence of neurofibrillary tangles [6] and focal atrophy [7, 8] in the medial temporal lobe (MTL). The loss of anterograde memory as a consequence of MTL lesions has been known since the description of the famous H.M. case [9], thus establishing the role of the MTL in the storage process [10]. More particularly, this is the case for episodic memory, which primarily consists of autobiographical memory, and is associated with a conscious state of recollection in a specific spatio-temporal context or autonoetic awareness [11]. In a clinical setting, the so-called ‘episodic’ memory is assessed using anterograde memory tests, which are widely used as a clinical marker of MTL dysfunction. Nevertheless, episodic memory does not solely include a storage process associated with the MTL. A task thought to evaluate episodic memory also requires other additional cognitive processes. These are the attentional and perceptual processes during the ‘encoding’ trial, which represents the initial registration phases of memory, as well as executive functions during ‘retrieval’ of the memory trace, which is the strategic effortful recollection phase. These processes are associated with extra-MTL aspects [12, 13]. Thus, a memory test aimed at localizing MTL dysfunction should be capable of distinguishing the different memory phases, namely storage and the encoding and retrieval processes.

Among the different verbal memory tests, the Free and Cued Selective Reminding Test (FCSRT) has been recommended by the International Working Group for the diagnosis of Alzheimer’s disease [2, 14]. This test appears to be particularly useful because it allows the different memory phases to be distinguished [15]. Indeed, initially designed by Grober and Buschke, the FCSRT is an anterograde verbal memory test based on semantic cueing, which allows controlling for the encoding process and facilitates retrieval [16]. This task therefore enables one to isolate the patients’ storage abilities and can be used to define an ‘amnestic syndrome of the medial temporal type’, which is characterized by a diminished free recall (FR) ability with no cueing enhancement; that is, impaired total recall (TR) [5, 17]. This test was found to be predictive for dementia [18], even in the very mild stages [19], and more particularly dementia of Alzheimer’s disease type [17], as compared with normal ageing [20], other forms of dementia [21, 22] or depression [23]. Moreover, performances on the FCSRT have proven useful to predict or exclude conversion to Alzheimer’s disease dementia in MCI patients [15, 19, 24, 25] or in individuals from a population-based study [26, 27]. Whereas TR proved more specific in distinguishing Alzheimer’s disease from other dementias [15], FR was more sensitive in predicting conversion to dementia of any type in a primary care cohort of MCI patients [19]. A decreased cued recall is also concordant with the existence of a CSF profile characteristic for Alzheimer’s pathology in MCI patients [28]. Moreover, the performances on the test were associated with the progression of neurofibrillary lesions within the MTL in a neuropathological study of Alzheimer’s disease patients [29]. In previous studies using neuroimaging in MCI or Alzheimer’s disease patients, performances on the FCSRT were already found to be correlated with MTL volume in structural imaging [30–32] or with its activity in resting state functional imaging [13], as well as in community older adults [33]. Various subregions of the MTL were implicated: hippocampus [30–32], parahippocampal cortex [13, 32], entorhinal/perirhinal cortex (in the MCI subgroup in [31, 32]), specifically on the left [31, 32] or bilaterally [13] (in the MCI subgroup in [31]). Performances on FR were related to prefrontal regions in one study [13]. Moreover, in a longitudinal study involving aMCI patients, those who had deficient scores on TR developed grey matter (GM) atrophy within the left anterior and lateral temporal lobe, whereas those who had deficient scores on FR only developed subcortical and frontal GM loss [34].

Besides contextualized ‘episodic’ memory associated with recollection, recognition memory has been described as a more implicit-like form of memory based on the sense of familiarity [35, 36]. Recognition memory has also been suggested as a potential early marker of Alzheimer’s disease in MCI patients because it relies on the MTL [37]. Contrary to episodic memory, recognition does not necessarily require a conscious state of recollection (‘remembering’) since a sense of familiarity (‘knowing’) is sufficient to recognize a target item between two paired items, suggesting two different cognitive processes for familiarity and recollection, as well as independent neuroanatomical substrates, as described in the dual-process model [35]; an alternative view involving memory strength has also been proposed [38]. Both animal experiments (e.g. in monkeys [39], in rodents [40]) and human case studies (e.g. [41, 42]) have shown that performances on visual recognition memory tasks were related to damage in the perirhinal cortex rather than in the hippocampus [35, 36]. Yet the perirhinal cortex (BA35) is the subregion within the MTL where neurofibrillary tangles initially appear in Alzheimer’s disease, before spreading to the entorhinal cortex and finally reaching the hippocampal formation [43]. Barbeau et al*.* [37] therefore developed a visual recognition memory test aimed at detecting Alzheimer’s disease in the earliest stages. The task consists of a visual delayed matching-to-sample task (the Delayed Matching-to-Sample—48 items (DMS-48)), which includes an implicit encoding phase during an immediate trial and a 1-hour delayed trial based on a forced-choice recognition. Impaired performances on the DMS-48 were found in aMCI, with intermediate scores between Alzheimer’s disease patients and controls, congruent with the presence of an ‘amnestic syndrome of the medial temporal type’ on the FCSRT [37]. The MCI patients who failed on the DMS-48 showed a pattern of GM loss on structural MRI [44] and hypoperfusion on single photon emission tomography [45] including the MTL and bilateral temporo-parietal regions, as opposed to prefrontal defect in the MCI patients who succeeded in this task. Such a temporo-parietal pattern usually being described in the early stages of Alzheimer’s disease (e.g. [46]), the authors [44] suggested that the DMS-48 allows patients at high risk for Alzheimer’s disease to be detected within a population of patients with MCI. In another study using magnetic resonance spectroscopic imaging, the same team showed that aMCI patients with impaired scores on the DMS-48 had metabolic changes within the MTL reflecting regional pathological changes [47]. Moreover, patients with impaired performance on the DMS-48 develop a typical pattern of cognitive profile as described for Alzheimer’s disease, and a prolonged clinical follow-up indicates that it is reliable marker to predict conversion to dementia [48, 49].

Given the implication of early MTL lesion in Alzheimer’s disease and the need for reliable diagnostic markers, the objective of this study was to evaluate the neuroanatomical correlates of the FCSRT and the DMS-48 using structural MRI on a large cohort of MCI patients. We were expecting the performances on the two tests to be correlated with GM volume in the MTL for specific profiles of memory indicating a ‘storage’ deficit; namely, when patients had impaired TR scores on the FCSRT, which is referred to as an ‘amnestic profile of the medial temporal type’ in the aforementioned literature, or when their scores declined on the delayed recall of the DMS-48, according to our clinical experience. Considering the classical view of a dual process model and the hemispheric lateralization, we were more particularly expecting the left hippocampus to be involved for the performances on the FCSRT and the right perirhinal cortex for the performances on the DMS-48. Conversely, we hypothesized that a deficit in strategic retrieval or in attentional/perceptual processes would respectively trigger impaired performances on FR for the FCSRT when the score normalizes with cueing and on the DMS-48 when the score does not decline between Set1 and Set2. Such a ‘retrieval’ or ‘encoding’ deficit profile would be related to extra-MTL regions.

## Methods

### Participants

The cohort consisted of patients who were consecutively included in a currently ongoing longitudinal, multicentre, clinical research study. From this population, we selected a subgroup of 138 patients from two centres (Department of Geriatrics of Broca Hospital in Paris, France, and the CMRR at the University Hospital of Strasbourg, France) where the patients were included and followed up. In their respective centres, the patients systematically underwent clinical examination, including cognitive assessment and a high-resolution MRI scan. The study was approved by the local ethics committee of Ile de France IV. All participants provided written informed consent.

All patients included in the present study were diagnosed with MCI [3], based upon a complete clinical examination and cognitive evaluation. Subsequently, the participants were categorized into aMCI and non-amnestic MCI (naMCI) subtypes [4]. These categories were further subdivided into single-domain (sd) and multi-domain (md) subtypes.

To be included, the patients had to be aged older than 70 years. A minimum of 4 years of education and a proficient level in French language were required, in order to avoid limitations during the cognitive assessment. Patients with additional neurological or psychiatric conditions, or medical diseases that impacted audition or vision and thus interfered with the cognitive evaluation, were excluded. Patients with contraindications to MRI were excluded, as well as left-handed patients for the purpose of distinguishing the hemispheric lateralization of verbal vs*.* visual memory performances during the voxel-based morphometry (VBM) analyses.

The patients and their informants underwent a clinical interview and an evaluation of daily functioning (CDR [50], IADL [51] and ADL scales [52]). The patients also underwent a complete clinical examination, a large battery of neuropsychological tests (Verbal Fluencies [53], DO80 [54], TMT A and B [55], Similarities, Digit symbol and Digit span subtests of the WAIS [56]) in addition to the MMSE [57] and to the two memory tasks (see infra), as well as screening for depression with the GDS [58]. Educational level (EL) was classified into seven categories with a scale proposed by Barbizet and Duizabot [59], ranging from EL3 (5 years) to EL7 (university studies) in our study. All participants underwent an MRI scan and standardized blood testing. The demographic and main clinical characteristics are summarized in Table 1.

**Table 1:** Demographic and main clinical data of the whole cohort

Age	79.1 (5.6)
Gender (male/female)	47/91
EL: number of patients depending on the category	EL3: 11; EL4: 34; EL5: 16; EL6: 19; EL7: 58
MCI subtypes: number of patients depending on the subtype	aMCI: 97 (sd: 20, md: 77)
	naMCI: 41 (sd: 35, md: 6)
MMSE	27.1 (1.7)
CDR: number of patients depending on the score	Score 0: 28
	Score 0.5: 110
IADL	13.1 (1.3)

Data presented as mean (standard deviation) unless stated otherwise

*aMCI* amnestic mild cognitive impairment, *naMCI* non-amnestic mild cognitive impairment, *MCI* mild cognitive impairment, *sd* single-domain, *md* multi-domain, *EL* educational level, *CDR* Clinical Dementia Rating Scale, *IADL* Instrumental Activity of Daily Living, *MMSE* Mini-Mental State Examination

### Anterograde memory tests

#### Free and Cued Selective Reminding Test

The FCSRT is a verbal memory test based on semantic cueing. This allows for controlling of encoding processes, and facilitates retrieval. During an encoding phase, 16 words are presented four by four and must be associated with a category cue. The subjects are then asked to recall the words on three successive trials, and then on a 30-minute delayed trial. Each trial includes FR and a cued recall, where the semantic category is provided for the items that were not spontaneously retrieved by the patient. The TR score represents the sum of FR and the cued recall on the three trials. Delayed total recall (DTR) refers to the TR score for the delayed trial (see detailed procedure in [16]).

In order to adjust the cut-off value to the age and EL in our French cohort, we used the norms for the FCSRT published by Amieva et al. [60]. Considering that FR would reflect retrieval ability, whereas only TR would truly reflect storage ability, we defined three groups with different memory profiles as follows: group A_1_ for patients with a storage or mixed storage and retrieval deficit (deficient TR scores); group B_1_ for patients with a pure retrieval deficit (deficient FR but normal TR); and group C_1_ for patients with normal scores on both FR and TR. The whole cohort of MCI patients will be referred as group ABC. See Table 2 for the performances of each group on the FCSRT. Note that group A_1_ was significantly younger than groups B_1_ and C_1_. No significant difference existed between the groups for the EL.

**Table 2:** Performances on the FCSRT and the DMS-48 according to the memory profile and demographic characteristics of the different groups

	Group			
Test	ABC	A_1/2_	B_1/2_	C_1/2_
FCSRT	*n* = 138	*n* = 50	*n* = 18	*n* = 70
Age	79.1 (5.6)	77.4 (5.5)^##^	83.5 (6.4)^#^	79.4 (4.7)
EL	5.6 (1.5)	5.6 (1.4)	5.5 (1.5)	5.6 (1.5)
FR	17.1 (8.8)	9.7 (5.7)*	10.5 (2.7)*	24.1 (5.2)
TR	37.8 (9.7)	28.8 (8.6)**	40.0 (4.1)*	44.8 (2.8)
DTR	13.0 (3.5)	9.4 (3.3)**	13.8 (2.0)*	15.3 (1.0)
DMS-48	*n* = 138	*n* = 36	*n* = 25	*n* = 77
Age	79.1 (5.6)	78.5 (6.3)	79.8 (5.3)	79.4 (5.2)
EL	5.6 (1.5)	5.6 (1.5)	5.6 (1.3)	5.5 (1.5)
Set1	43.2 (5.1)	40.9 (4.8)*	35.7 (5.3)*	46.3 (1.6)
Set2	42.6 (5.4)	36.7 (5.0)*	39.3 (4.7)*	46.2 (1.5)

Data presented as mean (standard deviation)

^#^ Significant difference with *P* < 0.05 compared with group C_1/2_

^##^ Significant difference with *P* < 0.05 compared with groups B_1/2_ and C_1/2_

* Significant difference with *P* < 0.001 compared with group C_1/2_

** Significant difference with *P* < 0.001 compared with groups B_1/2_ and C_1/2_

*DMS-48* Delayed Matching-to-Sample—48 items, *FCSRT* Free and Cued Selective Reminding Test, *EL* educational level, *FR* free recall score, *TR* total recall score, *DTR* delayed total recall score, *Set1* immediate score, *Set2* 1-hour delayed score

#### Delayed Matching-to-Sample—48 items

The DMS-48 is a visual forced-choice recognition memory test. It is based on a delayed matching-to-sample paradigm, where the subjects are being asked to choose between a target and a distractor. During an implicit encoding phase, the subjects are asked to decide whether they see more or fewer than three colours on 48 consecutive target items. The stimuli belong to three different categories: abstract pictures; concrete objects that belong to the same semantic category (e.g. two cats); and concrete objects that do not belong to the same category (e.g. carrot and cat). The two first categories allow the use of verbal strategies to be limited. After the encoding phase, an immediate and a 1-hour delayed recognition trial are proposed with two different sets of distractors, Set1 and Set2, respectively (see detailed procedure in [37]). We used the norms published by Barbeau et al. [37], which take age into account. In order to test our hypothesis, according to which only patients whose scores declined between Set1 and Set2 truly have a memory deficit related to MTL dysfunction (‘storage-like’ deficit), we defined three groups depending on the memory profile as follows: group A_2_ for patients with a ‘storage-like’ deficit (Set2 < Set1); group B_2_ for patients with ‘encoding’ deficit (Set2 ≥ Set1); and group C_2_ for patients with normal scores on both Set1 and Set2. The whole cohort of MCI patients will be referred to as group ABC. See Table 2 for the performances of each group on the DMS-48. Note that the groups of patients were different for the DMS-48 and the FCSRT. Note that no significant difference existed between the groups for age and EL.

### Neuroimaging study

We used VBM to investigate the neuroanatomical correlates of anterograde memory performances in the MCI patients. To map the regions of atrophy related to the memory deficit, we tested correlation with the GM volume at a voxel level with the scores on both memory tests in the patients. Each participant underwent a high-resolution anatomical MRI scan at inclusion. T1-weighted three-dimensional anatomical images were obtained using 3 T MRI scanners in Strasbourg (Verio 32-channel Tim Siemens scanner; Siemens, Erlangen, Germany) and in CENIR, ICM, Paris (Verio and Trio 32-channel Tim Siemens scanner; Siemens) using a volumetric Magnetization Prepared Rapid Acquisition with Gradient Echo (MPRAGE) sequence (FOV = 256 × 256 mm^2^, image matrix = 256 × 256, slice thickness = 1 mm; Strasbourg site: repetition time = 1900 ms, echo time = 2.52 ms, flip angle = 9°; Paris site: repetition time = 2300 ms, echo time = 4.18 ms, flip angle = 9°).

VBM analyses included image pre-processing and statistical analyses. These steps were carried out using the SPM12b software package (Wellcome Department of Imaging Neuroscience, London; http://www.fil.ion.ucl.ac.uk/spm) running on Matlab R2010a (MathWorks, Natick, MA, USA). Anatomical MRI images were spatially pre-processed using standard procedures [61]. All T1-weighted structural images were first segmented, bias corrected and spatially normalized to the Montreal Neurological Institute (MNI) space using an extension of the unified segmentation procedure [62] that includes six classes of tissue. The DARTEL registration toolbox was then used to build a study-specific template and to bring into alignment all of the segmentation images. The VBM analysis was done on modulated GM images; that is, the GM value in each voxel was multiplied by the Jacobian determinant derived from the spatial normalization. This procedure preserves the total amount of GM from the original images. These modulated GM images were smoothed with a Gaussian kernel (FWHM: 8 mm).

### Statistical analysis

#### Behavioural analyses

Intergroup differences between the demographic data and the memory scores on the two tests were compared using a Student’s *t* test.

#### VBM analyses

Statistical correlations between local GM volume and scores on both memory tests were then investigated using the General Linear Model (GLM). Raw scores on the FCSRT (FR, TR and DTR) and the DMS-48 (Set1 and Set2) were tested successively for groups A_1/2_, B_1/2_ and C_1/2_, pooled (group ABC) and independently, by entering each of them as a covariate of interest. The correlations were tested using *t* contrasts (one-tailed test), assuming that decreased memory performances would be associated with decreased GM volumes. Different nuisance covariates were considered in the model: the age of the subjects, EL, the total GM volume, and site of acquisition (because of two different MRI scans). We used a statistical threshold of *P* < 0.05 with family-wise error (FWE) as the correction for multiple analyses whenever possible. For each detected cluster, partial correlation analyses were conducted between the mean GM volume of the cluster and FCSRT or DMS-48 scores while taking into account the same set of nuisance covariates (results shown in Additional file 1: Figure S1). When no correlations were found using FWE, a less stringent statistical threshold of *P* < 0.001, uncorrected, was considered. A cluster spatial extent of 50 voxels was used in this case to avoid irrelevant and isolated detections, unless no correlation over 50 voxels was found at all. The software Xjview (http://www.alivelearn.net/xjview8/) allowed us to identify the brain regions and to determine the number of voxels within each region included in each cluster. In the present work, we refer to the hippocampus according to the AAL atlas, namely as the hippocampus proper plus the dentate gyrus and uncus [63]. Reference to the ‘MTL’ includes additionally the entorhinal (BA28 and BA34), perirhinal (BA35) and parahippocampal (BA36–BA37) cortices, which together constitute the parahippocampal gyrus [64]. We also performed group analyses to compare the GM volume between groups A_1/2_ vs*.* C_1/2_ and groups B_1/2_ vs*.* C_1/2_, using a *t* test, including the same nuisance covariates, in order to check whether the MTL would be atrophied in the group A_1/2_, in which the MTL was expected to be found in correlation analyses (results shown in Additional file 2: Figure S2).

## Results

### Free and Cued Selective Reminding Test

**Fig. 1:**
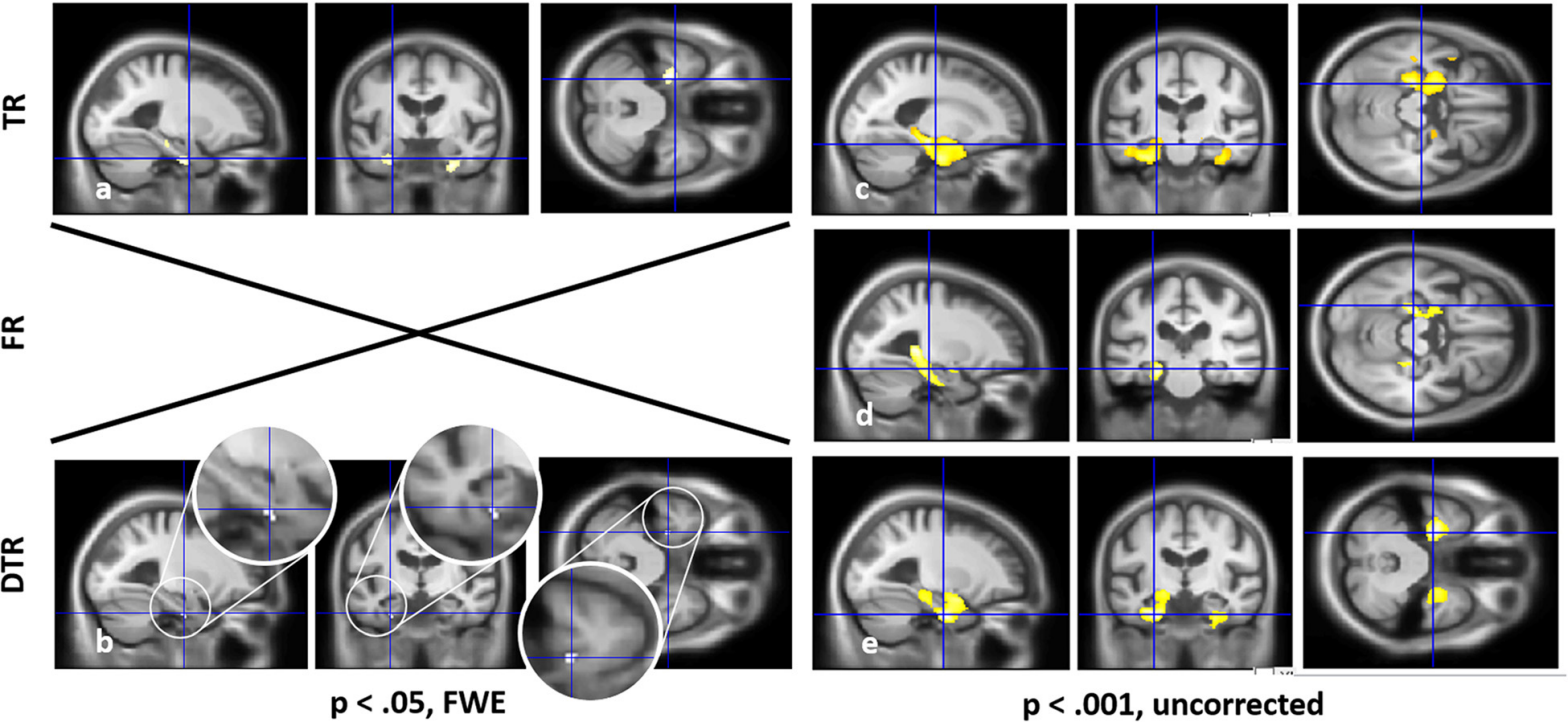
VBM analyses for the FCSRT in the whole group of patients (group ABC). GM volume regions positively correlated with the TR **a, c**, FR **d** and DTR **b, e**, including age, gender, EL, total GM volume and centre as nuisance covariates, with a threshold of *P* = 0.05, FWE **a, b** or *P* = 0.001, uncorrected **c–e**. *DTR* delayed total recall, *FR* free recall, *FWE* family-wise error, *TR* total recall

For group ABC (Fig. 1 and Table 3), VBM analyses on the whole cohort revealed correlations after FWE correction for the TR and DTR scores. For TR, the cluster mainly involved the parahippocampal gyrus bilaterally, more particularly the perirhinal cortex (BA35), and to a lesser extent the anterior hippocampus (Fig. 1a and Additional file 1: Figure S1.a–c). For DTR, only the left perirhinal cortex (BA35) was found after FWE correction (Fig. 1b and Additional file 1: Figure S1.d). With a less stringent threshold of *P* < 0.001, uncorrected, a larger correlation with the MTL (hippocampus, entorhinal cortex (BA28 and BA34), perirhinal (BA35), parahippocampal cortex (BA36)) appeared bilaterally for the TR, DTR and FR scores, and with the lateral temporal cortex for TR and DTR (Fig. 1c–e). In each case, the left clusters’ volumes were larger than those on the right.

**Table 3:** GM volume regions positively correlated with the scores on the FCSRT for the whole group of patients (group ABC) using VBM

Group (score)	*P*	Brain region	Side	BA	*k*	*x*	*y*	*z*	*T*
ABC (TR), *n* = 138	*P*_FWE_ = 0.05	Parahippocampal g.	L	35	212	–26	–15	–27	5.59
		Hippocampus		NA	33	–21	–27	–12	5.13
		Parahippocampal g.	R	35	154	26	–10	–32	5.48
	*P* = 0.001	Hippocampus	L	NA	1227/4337	–28	–10	–24	5.02
		Parahippocampal g.		28, 34–36	818/4337	–26	–15	–27	5.59
		Temporal pole		38	281/4337	–31	6	–21	4.25
		Temporal lobe		38	905/4337	–31	6	–21	4.25
		Amygdala		NA	372/4337	–28	–3	–21	4.23
		Parahippocampal g.	R	20, 28, 36	812/1700	27	–15	–33	4.56
		Hippocampus		NA	172/1700	–25	–9	–24	3.91
		Temporal pole		NA	20/53	37	13	–18	3.39
		Hippocampus		NA	99/1200	–25	–9	–24	3.91
ABC (FR), *n* = 138	*P* = 0.001	Hippocampus	L	NA	470/1188	–24	–34	–3	3.82
		Parahippocampal g.		28, 35	295/1188	–19	–28	–13	3.97
		Amygdala		NA	25/1188	–21	–9	–12	3.26
		Hippocampus	R	NA	264/572	25	–35	1	3.65
		Parahipocampal g.		27	104/572	21	–34	–3	3.76
ABC (DTR), *n* = 138	*P*_FWE_ = 0.05	Parahippocampal g.	L	35	25	–26	–15	–27	5.06
	*P* = 0.001	Parahippocampal g.	L	28, 34–36	1646/2401	–26	–15	–27	5.06
		Hippocampus		NA	711/2401	–27	–10	–24	4.38
		Temporal lobe		20	151/2401	–37	–12	–25	3.29
		Amygdala		NA	313/2401	–25	0	–18	3.65
		Parahippocampal g.	R	28, 35, 36	496/850	25	–12	–36	4.04
		Hippocampus		NA	36/850	27	–10	–25	3.61
		Temporal lobe		20	68/850	37	13	–18	3.39

Statistical analyses were performed including age, gender, education level, total GM volume and centre as nuisance covariates, with a threshold of *P* = 0.05, FWE or *P* = 0.001, uncorrected, including a minimal *k* of 50 voxels

*GM* grey matter, *FCSRT* Free and Cued Selective Reminding Test, *VBM* voxel-based morphometry, *L* left, *R* right, *BA* Brodmann area, k cluster size in voxel (specific region’s volume/cluster’s global volume), x, y, z Talairach coordinates, T *T* value, *g.* gyrus, *DTR* delayed total recall, *FR* free recall, *FWE* family-wise error, *TR* total recall

**Fig. 2:**
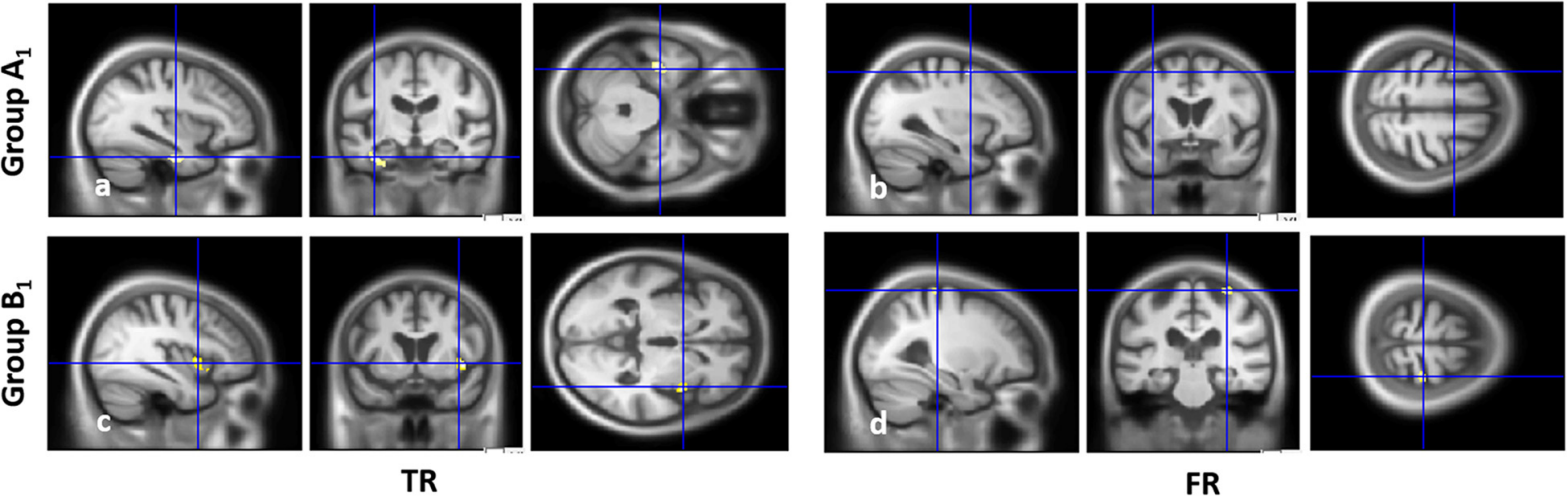
VBM analyses for the FCSRT depending on the memory profile. GM volume regions positively correlated with the scores on the FCSRT independently for group A_1_ **a, b** and group B_1_ **c, d**, for TR **a, c** (*left*) and FR **b, d** (*right*), including age, gender, EL, total GM volume and centre as nuisance covariates, with a threshold of *P* = 0.001. *FR* free recall, *TR* total recall

For groups A_1_ and B_1_ independently (Fig. 2 and Table 4), no correlations were found when VBM analyses were performed independently for groups A_1_, B_1_ and C_1_ with FWE correction; we therefore used a less stringent threshold of *P* < 0.001, uncorrected. VBM analyses then only revealed correlations with the MTL (perirhinal (BA35) and parahippocampal cortices (BA36)) and with the lateral temporal neocortex (fusiform gyrus) for TR for group A_1_ (Fig. 2a). The cluster was left-sided and mainly included parahippocampal gyrus (BA35 and BA36) and, to a lesser extent, hippocampus (see Table 4). Conversely, TR scores were correlated with insular cortex volume for group B_1_ (Fig. 2c) but not with the MTL. Finally, performances on FR were correlated with prefrontal aspects for both groups A_1_ (Fig. 2b) and B_1_ (Fig. 2d). In the case of group A_1_, the cluster size was smaller than 50 voxels, although we chose to take it into account since it was the only correlation found. Note that when comparing the cerebral volume of groups A_1_ and B_1_ vs*.* C_1_, the MTL was found to be atrophied bilaterally in group A_1_ only (*P* < 0.001, uncorrected; see Additional file 2: Figure S2).

**Table 4:** GM volume regions positively correlated with the scores on the FCSRT independently for groups A_1_ and B_1_ using VBM

Group (score)	*P*	Brain region	Side	BA	*k*	*x*	*y*	*z*	*T*
A_1_ (TR), *n* = 50	0.001	Parahippocampal g.	L	35/36	50/283	–28	–15	–28	3.62
		Hippocampus		NA	16/283	–34	–16	–21	3.47
		Fusiform g.		20	135/283	–37	–18	–24	3.68
A_1_ (FR), *n* = 50	0.001	Mid. Frontal g.	L	NA	16	–31	–1	49	3.41
B_1_ (TR), *n* = 18	0.001	Insula	R	13	88/170	42	3	0	4.38
B_1_ (FR), *n* = 18	0.001	Precentral g.	R	4	62/63	28	–30	60	5.60

Statistical analyses were performed including age, gender, education level, total GM volume and centre as nuisance covariates, with a threshold of *P* = 0.001, uncorrected, including a minimal *k* of 50 voxels except for the FR in group A_1_

*GM* grey matter, *FCSRT* Free and Cued Selective Reminding Test, *VBM* voxel-based morphometry, *L* left, *R* right, *BA* Brodmann area, k cluster size in voxel (specific region’s volume/cluster’s global volume), x, y, z Talairach coordinates, T *T* value, *g.* gyrus, *FR* free recall, *TR* total recall

### Delayed Matching-to-Sample—48 items

**Fig. 3:**
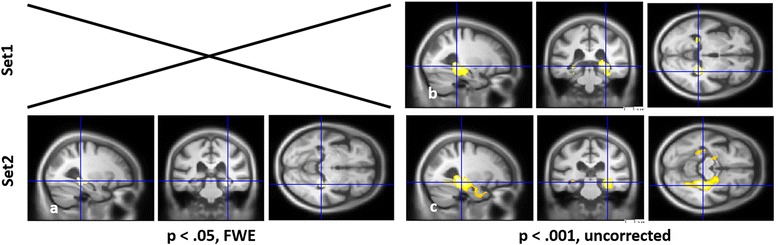
VBM analyses for the DMS-48 in the whole group of patients (group ABC). GM volume regions positively correlated with Set1 **b** and Set2 **a, c**, including age, gender, EL, total GM volume and centre as nuisance covariates, with a threshold of *P* = 0.05, FWE **a** or *P* = 0.001, uncorrected **b, c**. *FWE* family-wise error, *Set1* immediate score, *Set2* 1-hour delayed score

For group ABC (Fig. 3 and Table 5), VBM analyses on the whole cohort revealed correlations after FWE correction only for the scores on Set2 of the DMS-48, which exclusively involved the right posterior hippocampus (Fig. 3a and Additional file 1: Figure S1.e). With a less stringent threshold of *P* < 0.001, uncorrected, a larger correlation with the MTL appeared for both Set1 and Set2 (Fig. 3b, c), bilaterally but more prominently on the right, including hippocampus as well as parahippocampal gyrus (parahippocampal cortex (BA36 and BA37), perirhinal cortex (BA35), posterior entorhinal cortex (BA28) and BA19). The precuneus was also involved for Set1.

**Table 5:** GM volume regions positively correlated with the score on the DMS-48 for the whole group of patients (group ABC) using VBM

Group (score)	*P*	Brain region	Side	BA	*k*	*x*	*y*	*z*	*T*
ABC (Set1), *n* = 138	*P* = 0.001	Hippocampus	R	NA	606/1377	27	–34	–1	4.31
		Parahippocampal g.		28, 35–37	557/1377	28	–39	–6	4.36
		Hippocampus	L	NA	67/264	–28	–40	0	3.47
		Parahippocampal g.		19, 31	38/264	–27	–45	–10	3.41
		Precuneus		31	70/70	–2	–71	21	3.43
ABC (Set2), *n* = 138	*P*_FWE_ = 0.05	Hippocampus	R	NA	370	28	–37	–3	5.42
		Parahippocampal g.		36–37	370	27	–37	–9	5.33
	*P* = 0.001	Hippocampus	R	NA	1303/3820	30	–25	–12	4.65
		Parahippocampal g.		19, 28, 35–37	834/3820	22	–19	–22	4.02
		Hippocampus	L	NA	94/279	–25	–39	–3	3.67

Statistical analyses were performed including age, gender, education level, total GM volume and centre as nuisance covariate, with a threshold of *P* = 0.05, FWE or *P* = 0.001, uncorrected, including a minimal *k* of 50 voxels

*GM* grey matter, DMS-48, Delayed Matching-to-Sample—48 items, *VBM* voxel-based morphometry, *L* left, *R* right, *BA* Brodmann area, k cluster size in voxel (specific region’s volume/cluster’s global volume), x, y, z Talairach coordinates, T *T* value, *g.* gyrus, *FWE* family-wise error, *Set1* immediate score, *Set2* 1-hour delayed score

**Fig. 4:**
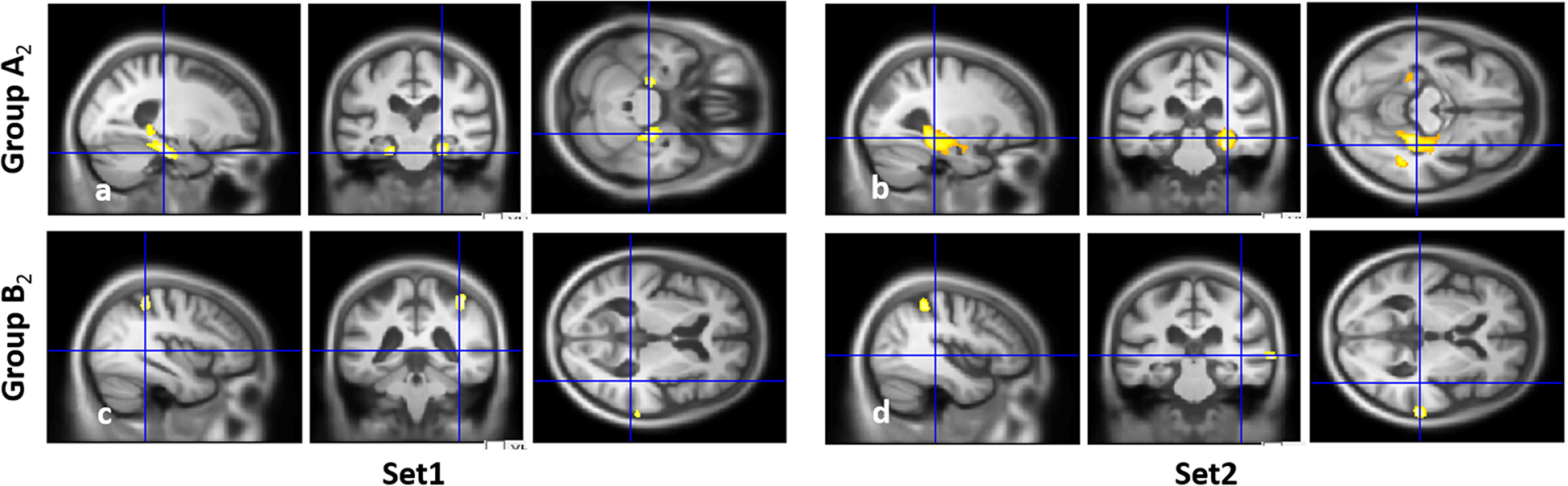
VBM analyses for the DMS-48 depending on the memory profile. GM volume regions positively correlated with the scores on the DMS-48 independently for group A_2_ **a, b** and group B_2_ **c, d**, for Set1 **a, c** (*left*) and Set2 **b, d** (*right*), including age, gender, EL, total GM volume and centre as nuisance covariate, with a threshold of *P* = 0.001. *Set1* immediate score, *Set2* 1-hour delayed score

For groups A_2_ and B_2_ independently (Fig. 4 and Table 6), when VBM analyses were performed using FWE correction only one correlation with the right hippocampus was found for group A_2_ with Set2 (see Table 6). We therefore used a less stringent threshold of *P* < 0.001, uncorrected. VBM analyses then only revealed correlations with the MTL (hippocampus, parahippocampal cortex (BA36–BA37), perirhinal cortex (BA35) and posterior entorhinal cortex (BA28)) for group A_2_ both with Set1 and Set2 (Fig. 4a, b). The clusters were bilateral but larger on the right, and prominently included parahippocampal gyrus for Set1 and hippocampus for Set2, when considering both the volume and *T* value (Table 6). Conversely, scores on Set1 and Set2 were correlated with temporal and parietal volumes for group B_2_ (Fig. 4c, d) but not with the MTL. Note than when comparing the cerebral volume of groups A_2_ and B_2_ vs. C_2_, respectively, the MTL was found to be atrophied in group A_2_ only, specifically on the right (*P* < 0.005, uncorrected; see Additional file 2: Figure S2).

**Table 6:** GM volume regions positively correlated with the score on the DMS-48 independently for groups A_2_ and B_2_ using VBM

Group (score)	*P*	Brain region	Side	BA	*k*	*x*	*y*	*z*	*T*
A_2_ (Set1), *n* = 36	*P* = 0.001	Parahippocampal g.	R	28, 35–37	493/907	24	–27	–20	5.00
		Hippocampus		NA	190/907	28	–38	–1	4.26
		Parahippocampal g.	L	28, 35	194/211	–24	–27	–20	4.35
A_2_ (Set2), *n* = 36	*P*_FWE_ = 0.05	Hippocampus	R	NA	25	27	–34	–12	6.59
	*P* = 0.001	Hippocampus	R	NA	1091/2669	30	–33	–7	5.05
		Parahippocampal g.		28, 35, 36	776/2669	24	–36	–15	3.78
		Parahippocampal g.	L	28, 35	260/381	–18	–17	–22	5.04
		Hippocampus		NA	79	–21	–15	–20	3.49
B_2_ (Set1), *n* = 25	*P* = 0.001	Inf. parietal lobule	R	40	153	37	–43	45	4.28
		Sup. Temporal g.		22	80	66	–35	1	4.61
		Inf. Temporal g.	L	20	101	–51	–15	–34	4.08
		Sup. Temporal g.		22	54	–60	–48	–10	4.73
B_2_ (Set2), *n* = 25	*P* = 0.001	Sup. Temporal g.	R	22	179	66	–33	1	5.98
		Inf. parietal lobule		40	161	37	–43	45	5.46

Statistical analyses were performed including age, gender, education level, total GM volume and centre as nuisance covariate, with a threshold of *P* = 0.05, FWE or *P* = 0.001, uncorrected, including a minimal *k* of 50 voxels

*GM* grey matter, DMS-48, Delayed Matching-to-Sample—48 items, *VBM* voxel-based morphometry, *L* left, *R* right, *BA* Brodmann area, k cluster size in voxel (specific region’s volume/cluster’s global volume), x, y, z Talairach coordinates, T *T* value, *g.* gyrus, *FWE* family-wise error, *Set1* immediate score, *Set2* 1-hour delayed score

## Discussion

In the present study, we investigated the neuroanatomical correlates of two anterograde memory tests, the FCSRT and the DMS-48, in a cohort of MCI patients using VBM. Overall, we found that the scores on the two memory tests were correlated with the volume of the MTL, prominently on the left side for the FCSRT and on the right side for the DMS-48, concordant with the respective verbal and visual modality of the two tests. However, when analysing different subgroups of patients according to their memory profile, we showed that a correlation with the MTL existed only in patients with a deficient TR score on the FCSRT. Similarly, a correlation with the MTL existed only when considering the subgroup of patients with worsening performances between Set1 and Set2 of the DMS-48. The present study confirms that both tests are reliable topographical markers, when properly interpreted, to indicate a profile of the MTL. Partially contradicting our hypothesis, the parahippocampal gyrus was prominently involved for both the visual recognition memory task and the verbal memory task supposed to assess ‘episodic’ memory, whereas the hippocampus was prominently involved for the delayed recall of the visual recognition memory task. Our conclusions are based on correlational analysis rather than group analysis since we did not include a control group without memory complaints, which is one limitation of the study. This finally allowed us to unravel more precisely the different memory phases and the contribution of their cerebral substrates.

### Neuroanatomical correlates of the FCSRT

Regarding the FCSRT, our study confirms in a larger cohort the results of previous studies showing the implication of the MTL in MCI or Alzheimer’s disease [13, 30–32]. As expected [32], implication of the MTL was found for the TR scores, but was also the case for the FR scores when considering the whole group of patients (for similar results with other memory tests, see also [12, 65–67]). Within the MTL, we found a correlation with the parahippocampal gyrus, which was stronger for the perirhinal cortex (BA35), encompassing the entorhinal cortex [31, 32] (see also [67–69]), and the parahippocampal cortex [13, 32], as well as a correlation with the hippocampus [30–32] (see also [12, 65, 70, 71]). These results are consistent with the involvement of the MTL in the consolidation and storage of new information [10, 36]. This correlation existed prominently on the left, as reported previously with the FCSRT [31, 32] and other tests [12, 66, 68], consistent with the verbal modality [66, 72, 73].

Interestingly, we were able to distinguish the neuroanatomical correlates of the group of patients who had low performances on TR (group A_1_) from those who had deficient FR scores only (group B_1_). Thus, we found that MTL atrophy existed only in group A_1_ and correlated with their deficient TR scores, reflecting the existence of a storage deficit. We therefore confirmed that the memory profile with few cueing improvements could indeed be considered an ‘amnestic syndrome of the medial temporal type’ [5, 17]. The correlation obtained with the MTL for the whole group was probably driven by group A_1_, since no correlation was found with the MTL in group B_1_. Regarding the MTL subregions found, when considering the results obtained for group A_1_ or after FWE correction, the parahippocampal gyrus, including the perirhinal cortex, was more prominently involved than the hippocampus for the TR scores. In other words, our results suggest that the parahippocampal gyrus rather than the hippocampus allows the storage of ‘episodic’ memory, which contradicts the prediction of the dual process model [35, 36]. In this model, the storage of contextualized information associated with recollection is supposed to involve the hippocampus. Even though the FCSRT is based on recollection rather than familiarity, it is questionable to consider that strong contextual information is associated with such laboratory memory. Indeed, the retrieval of learned word lists is not comparable with real-life episodic memory such as autobiographical memory [74], which indeed involves the hippocampus (e.g. [75–77]). Moreover, cued recall performances on the FCSRT were also found to be associated with the parahippocampal region by Lekeu et al. [13], which was interpreted as reflecting the specific semantic process associated with cueing (e.g. [78, 79]).

Finally, we found correlations involving extra-MTL regions, either for the TR scores in group B_1_ or for the FR scores in both groups A_1_ and B_1_. TR scores in group B_1_ were associated with the volume of the right insula, the significance of this correlation being limited by the fact that the TR scores were not deficient and no focal atrophy was found in group analyses. The FR scores in both groups A_1_ and B_1_ were associated with prefrontal aspects, as was the case in Lekeu et al.’s study [13], concordant with the implication of search activity and strategic retrieval of the information during FR [80]. Concordant with the pattern of regions associated with the two different memory profiles, in a longitudinal study involving MCI patients [34], those who had deficient scores on TR of the FCSRT developed GM atrophy within the left temporal lobe, whereas those who had deficient scores on FR only developed subcortical and frontal GM loss. Additionally, a correlation was also found for the whole group with the lateral temporal cortex, probably reflecting semantic aspects associated with verbal memory [81].

### Neuroanatomical correlates of the DMS-48

As expected, the MTL volume also proved to be correlated to the performances on the DMS-48, concordant with previous studies [44, 45, 47]. Namely, the implication of the MTL encompasses both the parahippocampal gyrus (including the entorhinal, perirhinal and parahippocampal cortex) and the hippocampus—although when the analyses were performed independently for each group, the implication of the MTL remained for group A_2_ only. In this group, performances declined between the immediate and delayed recall, which can therefore also be considered an ‘amnestic profile of the medial temporal type’, concordant with our hypothesis. Taken together with these previous imaging studies [44, 45, 47] and clinical longitudinal studies [48, 49], our results confirm that the DMS-48 constitutes an interesting clinical marker for potential Alzheimer’s disease pathology, with our additional contribution about the interpretation of the memory profile, which should decline over time to be suspected of MTL dysfunction. Additionally, the implication of the MTL was prominently right-sided (or exclusively right-sided for Set2 in group A_2_ after FWE correction), as was the case in a previous study [44], which is concordant with the visual modality of the task [66, 72, 73]. This shows that the test was particularly well designed to avoid the use of verbal strategy.

We will further consider the results obtained with group A_2_, which had an ‘amnestic profile of the medial temporal type’, to analyse more precisely the MTL subregions involved. Whereas the correlation with the MTL prominently involved the parahippocampal gyrus, namely the perirhinal cortex, over the hippocampus for immediate recall, the opposite pattern was found for delayed recall. In this case, the hippocampus alone was found after FWE correction. The involvement of the parahippocampal gyrus is widely demonstrated for recognition memory [35], with the perirhinal cortex being more particularly associated with object recognition, and the parahippocampal cortex with spatial information, while the hippocampus would bind item and contextual information [36]. In human studies, the right hippocampus was found to be associated with spatial navigation [82, 83] or with the visuo-spatial component of autobiographical memory [84]. In our study, the switch from parahippocampus to hippocampus during delayed recall was unexpected insofar as recognition memory was involved. This could reflect the existence of a deeper encoding of the memory trace due to a double registration of items after the first trial [38]. In particular, we cannot exclude that it is due to the fact that the encoding is no longer implicit once the subject realized he/she was performing a memory task on the first trial and sees the target items for the second time. Moreover, one can hypothesize that the recognition of items could be associated with a sense of recollection during the second trial and therefore involve the hippocampus [36]. Although this could be the case during an activation study, the results obtained in a correlation study probably reflect the minimal memory ability of the patient, namely a sense of familiarity. An R/K paradigm [85] should ideally have been included during the assessment of recognition memory to control for this parameter. Additionally, our results could indicate that the hippocampus is involved in the retention of information after a 1-hour delay, even when recognition memory is concerned.

Finally, performances of group B_2_ were correlated with extra-MTL regions, namely the temporal and parietal cortices. The implication of the inferior temporal gyrus probably reflects the process of visual identification through the visual ventral pathway [86, 87]. Moreover, visual attentional processes are probably supported by the inferior parietal lobule [88, 89], as well as by the superior temporal gyrus according to some authors [90, 91]. Similarly, the precuneus, which was also implicated for Set1 in the whole group, is thought to be involved in attention shift between object features [92, 93]. Thus, lower performances on the first trial probably reflect perceptual and attentional processes involved for encoding during the registration phase. Overall, our study suggests that recognition memory tests involving a unique trial might not be reliable enough to indicate MTL dysfunction because they do not allow a perceptual or attentional deficit to be distinguished from a memory deficit per se.

### Factors for the involvement of MTL subregions in memory

Altogether, we found the prominent involvement of the parahippocampal gyrus for the FCSRT and for the immediate trial of the DMS-48, whereas the hippocampus was prominently involved for the 1-hour delayed recall of the DMS-48. Considering the classical view of a dual process model distinguishing recollection vs*.* familiarity-based memory, respectively involved in contextualized memory and recognition memory [35, 36], the implication of the parahippocampus was expected for the DMS-48 but we would have expected the implication of the hippocampus for the FCSRT. Conversely, the parahippocampus was prominently involved for ‘episodic’ memory as assessed by the FCSRT and the hippocampus for the delayed recall of the DMS-48, even though recognition memory was involved. Therefore, our results cannot be explained by the different level of awareness between familiarity and recollection. Our results also contradict the hypothesis that a deeper encoding process, such as semantic judgment, would involve the hippocampus because it triggers a stronger memory trace than a shallower encoding process, such as perceptual judgment [38, 94]. Finally, the fact that the task consists of explicit or implicit encoding neither explains our results, since the FCSRT involves the parahippocampus even though the instruction to memorize is explicit.

Most interestingly, the implication of the hippocampus for the delayed recall of the DMS-48 suggests that it might be triggered by the duration of the memory trace. To the best of our knowledge, recognition memory tasks in humans do not usually involve such a delayed trial, making our results difficult to compare. However, this hypothesis is consistent with some animal studies with hippocampal damage [95, 96], in which recognition memory was found to be impaired after long retention delays [38]. According to this hypothesis of a time dependency, the hippocampus could also be expected for the delayed recall of the FCSRT but the delay involved in this case is shorter. A 1-hour delayed trial should ideally have been included. However, in support of this hypothesis, another study involving correlational analysis with MCI patients [31] showed that the entorhinal cortex was correlated with the performances on the FCSRT during the learning phase, and the left hippocampus was additionally involved for delayed recall. Similarly, Schmidt-Wilcke et al. [67] found performances on the CERAD to be correlated with the hippocampus for delayed recall and with the entorhinal/perirhinal cortex for immediate recall, suggesting the implication of short-term memory during the encoding phase [97]. However, demonstrating the persistence of such a correlation during the learning phase in our study would favour its implication beyond the encoding phase. Hence, we suggest that there might be a sequential involvement of the parahippocampus and the hippocampus, independent of the ‘episodic’ vs*.* recognition nature of memory, with the parahippocampus being preferentially involved during the encoding/learning phases and the hippocampus during storage after a 30-minute delay. This temporal sequence would be consistent with the anatomical organization of the MTL, with the different parahippocampal subregions constituting entry into the hippocampus [98]. This is also consistent with the existence of an accelerated forgetting in patients with hippocampal sclerosis, whose performances are normal on immediate recall but decline after a 1-hour delay [99].

## Conclusions

Our study confirms the interest in the FCSRT and the DMS-48 as topographical markers of the MTL, therefore constituting pertinent clinical markers for potential underlying incipient Alzheimer’s disease pathology. However, we have confirmed in a large cohort of MCI patients that solely the existence of deficient scores after cueing on the FCSRT constitutes an amnestic profile of the MTL type, whereas FR is associated with prefrontal aspects due to strategic retrieval processes. In a similar vein, our study shows that solely declining scores on recognition memory implicate the MTL, whereas attentional and perceptual processes are involved when the scores improve, highlighting the contribution of a delayed trial to interpret the performances on a recognition task. These tasks also allow a reliable assessment of lateralization since the anatomical correlates are prominently left-sided for the FCSRT and right-sided for the DMS-48, concordant with the respective verbal and visual modality of the two tests. When analysing the MTL subregions involved, our results suggest a sequential involvement of the parahippocampus and the hippocampus in time, with the hippocampus being prominently involved after a prolonged delay, independent of the existence of a sense of recollection or familiarity associated with the memory, and of the encoding strength. This study could be complemented by the inclusion of a region-of-interest analysis to directly compare the involvement of the hippocampus and the parahippocampus, as well as by the use of amyloid biomarkers to assess the diagnostic value for the underlying pathology.

## Additional files

Additional file 1: Figure S1. Showing partial correlations between the normalized behavioural scores and the normalized volumes of the clusters: normalized scores were obtained taking into account nuisance covariates (i.e. age of the subjects, EL, total GM volume, site of acquisition) for the mean FCSRT (Fig. **S1.a-c** for TR and **S1.d.** for DTR) or DMS-48 scores (Fig. **S1.e.**) and the mean GM volume of the clusters found using FWE correction in the whole group of patients (group ABC). (TIF 278 kb) Additional file 2: Figure S2. Showing group analyses: local GM loss in group A_1_ **S2.a** and group B_1_ **S2.b** as compared with group C_1_ and in group A_2_ **S2.c** and group B_2_ **S2.d** as compared with group C_2_ including age, gender, EL, total GM volume and centre as nuisance covariates, with a threshold of *P* = 0.001 **S2.a, S2.b, S2.d** or *P* = 0.005 **S2.c**, uncorrected. (TIF 709 kb)

## Abbreviations

aMCI: amnestic mild cognitive impairment
DMS-48: Delayed Matching-to-Sample—48 items
DTR: delayed total recall
EL: educational level
FCSRT: Free and Cued Selective Reminding Test
FR: free recall
FWE: family-wise error
GM: grey matter
MCI: mild cognitive impairment
md: multi-domain
MTL: medial temporal lobe
naMCI: non-amnestic mild cognitive impairment
sd: single-domain
TR: total recall
VBM: voxel-based morphometry
