# Supplementary figures and images for: Exploring anterograde memory: a volumetric MRI study in patients with mild cognitive impairment
Source: Alzheimers Res Ther. 2016 Jul 30;8:26. doi: 10.1186/s13195-016-0190-1 (PMC4967326; doi:10.1186/s13195-016-0190-1)

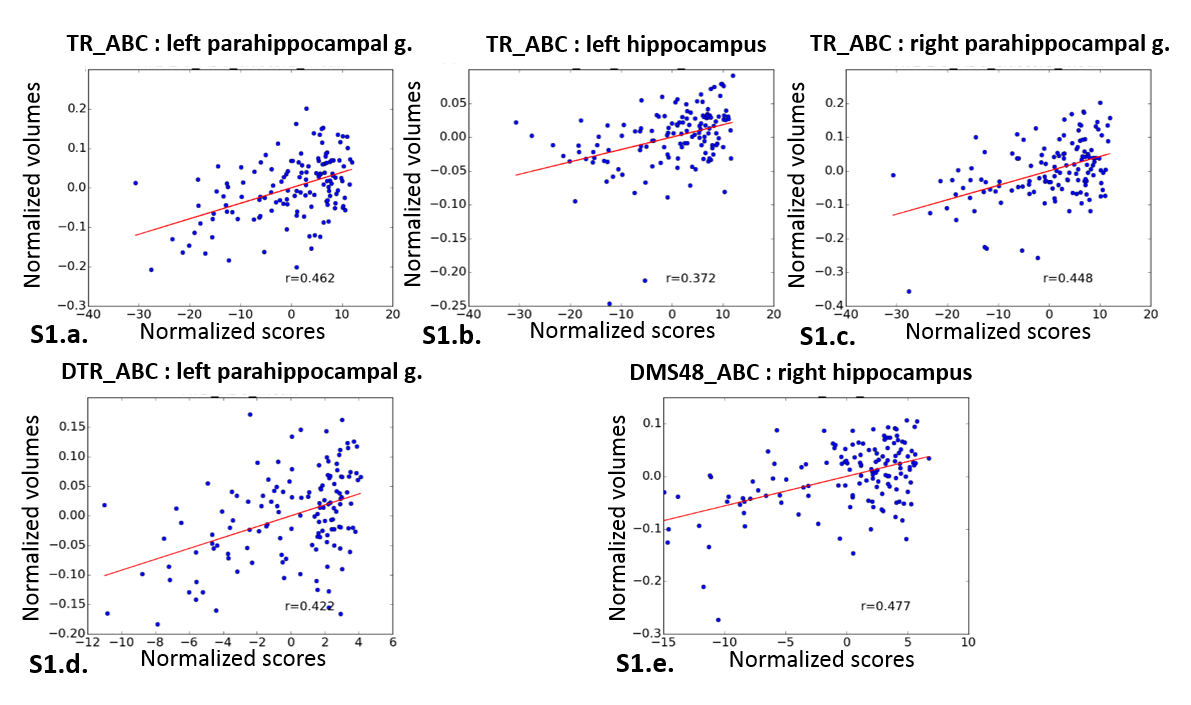

Supplement: Additional file 1: Figure S1. — Showing partial correlations between the normalized behavioural scores and the normalized volumes of the clusters: normalized scores were obtained taking into account nuisance covariates (i.e. age of the subjects, EL, total GM volume, site of acquisition) for the mean FCSRT (Fig. S1.a-c for TR and S1.d. for DTR) or DMS-48 scores (Fig. S1.e.) and the mean GM volume of the clusters found using FWE correction in the whole group of patients (group ABC). (TIF 278 kb) [file 13195_2016_190_MOESM1_ESM.tif]

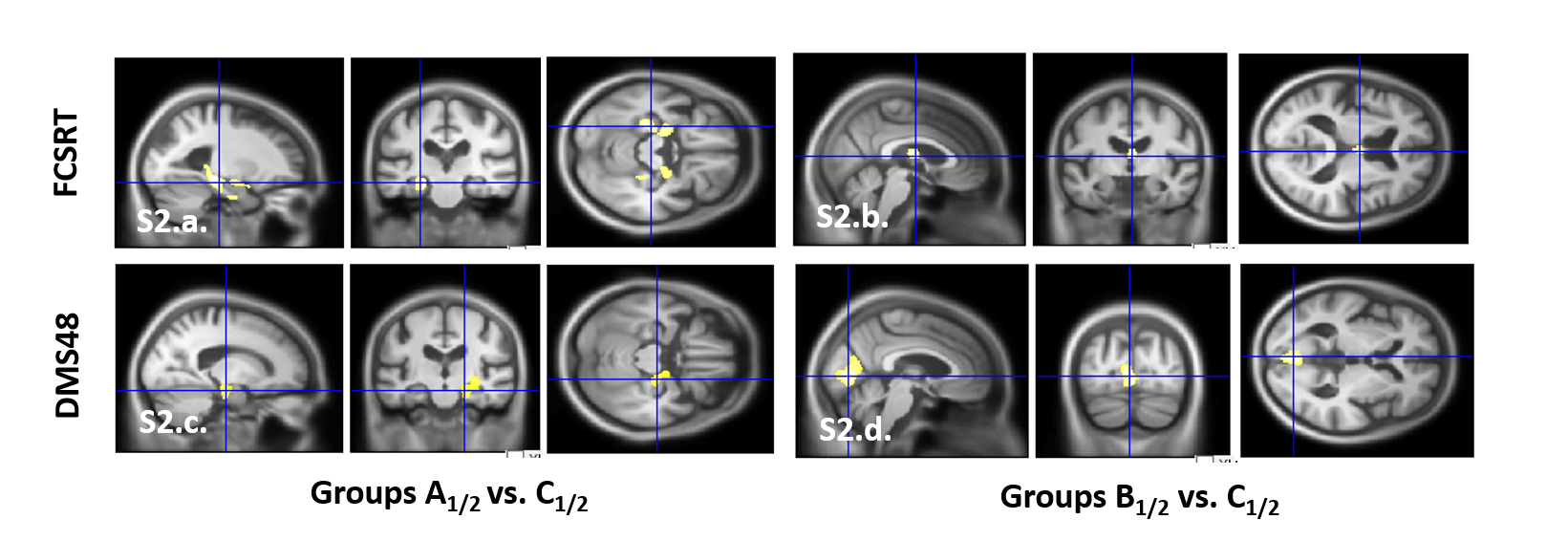

Supplement: Additional file 2: Figure S2. — Showing group analyses: local GM loss in group A1 S2.a and group B1 S2.b as compared with group C1 and in group A2 S2.c and group B2 S2.d as compared with group C2 including age, gender, EL, total GM volume and centre as nuisance covariates, with a threshold of P = 0.001 S2.a, S2.b, S2.d or P = 0.005 S2.c, uncorrected. (TIF 709 kb) [file 13195_2016_190_MOESM2_ESM.tif]
